# The professional radiation workforce in the United States

**DOI:** 10.1002/acm2.13848

**Published:** 2023-01-27

**Authors:** Wayne D. Newhauser, Jacqueline P. Williams, Michael A. Noska, Caridad Borrás, E. Vincent Holahan, Shaheen A. Dewji, Thomas E. Johnson, Jerry W. Hiatt, John W. Poston, Nolan Hertel, Dustin A. Gress, Michael D. Mills, David W. Jordan, Steven G. Sutlief, Melissa C. Martin, Edward Jackson, Edward I. Bluth, Donald P. Frush, M. Elizabeth Oates, Jeanne LaBerge, Hubert Y. Pan, Seth A. Rosenthal, Lawrence W. Townsend, Lori Brady, Janice Lindegard, Howard L. Hall, Elizabeth McAndrew‐Benavides, Eric Abelquist, Mitchell S. Anscher, Marcelo Vazquez, Amy Kronenberg, Jeffrey S. Willey, Theodore Lawrence, Gayle E. Woloschak, Brian Marples, Rosemary Wong, Michael Story, Roger W. Howell, Tom K. Hei, Sergey Y. Tolmachev, John D. Auxier, Thomas L. Rucker, Mikael Nilsson, Ralf Sudowe, Brian A. Powell, Mark P. Jensen

**Affiliations:** ^1^ Department of Physics and Astronomy Louisiana State University and Mary Bird Perkins Cancer Center Baton Rouge Louisiana USA; ^2^ University of Rochester Medical Center Rochester New York USA; ^3^ U.S. Food and Drug Administration Silver Spring Maryland USA; ^4^ Georgetown University Washington DC USA; ^5^ U.S. Nuclear Regulatory Commission Washington DC USA; ^6^ Georgia Institute of Technology Nuclear and Radiological Engineering and Medical Physics Programs George W. Woodruff School of Mechanical Engineering Atlanta Georgia USA; ^7^ Colorado State University Fort Collins Colorado USA; ^8^ Nuclear Energy Institute Washington DC USA; ^9^ Texas A&M University 3133 TAMU College Station Texas USA; ^10^ Georgia Institute of Technology North Avenue Atlanta Atlanta Georgia USA; ^11^ American College of Radiology Reston Virginia USA; ^12^ James Graham Brown Cancer Center Louisville Kentucky USA; ^13^ University Hospitals Cleveland Medical Center Cleveland Ohio USA; ^14^ Banner MD Anderson Cancer Center Phoenix Arizona USA; ^15^ Therapy Physics Inc. Gardena California USA; ^16^ University of Wisconsin Madison Wisconsin USA; ^17^ Ochsner Clinic Foundation New Orleans Louisiana USA; ^18^ Childrens Health Center Duke University Durham North Carolina USA; ^19^ University of Kentucky Lexington Kentucky USA; ^20^ University of California San Francisco California USA; ^21^ Sutter Radiation Oncology Center Sacramento California USA; ^22^ Sutter Radiation Oncology Center Roseville California USA; ^23^ University of Tennessee Knoxville Tennessee USA; ^24^ Nuclear Energy Institute Washington DC USA; ^25^ American Nuclear Society La Grange Park Illinois USA; ^26^ University of Tennessee Knoxville Tennessee USA; ^27^ Electric Power Research Institute Palo Alto California USA; ^28^ Oak Ridge Associated Universities Oak Ridge Tennessee USA; ^29^ Virginia Commonwealth University School of Medicine Richmond Virginia USA; ^30^ Loma Linda University Loma Linda California USA; ^31^ Lawrence Berkeley National Laboratory Berkeley California USA; ^32^ Wake Forest School of Medicine Winston‐Salem North Carolina USA; ^33^ University of Michigan ‐ University Hospital Ann Arbor Michigan USA; ^34^ Northwestern University Chicago Illinois USA; ^35^ National Cancer Institute Rockville Maryland USA; ^36^ The University of Texas Southwestern Dallas Texas USA; ^37^ Rutgers New Jersey Medical School Newark New Jersey USA; ^38^ Columbia University New York New York USA; ^39^ Washington State University Richland Washington USA; ^40^ University of Tennessee Knoxville Tennessee USA; ^41^ Leidos Reston Virginia USA; ^42^ University of California Irvine California USA; ^43^ Colorado State University Fort Collins Colorado USA; ^44^ Clemson University Anderson South Carolina USA; ^45^ Colorado School of Mines Golden Colorado USA

A radiation workforce of sufficient size and capacity is necessary to meet our nation's current and future needs for energy production, health care, and other vital areas. Over the long term, workforce shortages have the potential to compromise our nation's capabilities in these strategic sectors and, if sustained, would result in degradation of economic competitiveness and national security. In 2015, a multidisciplinary team began reviewing a selection of professional radiation workforces in the United States with the goal of developing a resource that would contain information of relevance to employers, policy makers, educational institutions, students contemplating radiation‐related careers, and the public. This approach was taken because ionizing radiation is used for a wide array of applications, and these frequently involve multidisciplinary teams. Indeed, the various radiation disciplines comprise a synergistic ecosystem, with many interdependencies, and this motivated us to review disciplines individually, as well as in the context of the larger multidisciplinary ecosystem.

The team members were drawn from those professions that are chiefly responsible for the radiation protection of workers, patients, and the public: health physics, medical physics, medicine (including diagnostic radiology, interventional radiology, nuclear medicine, and radiation oncology), nuclear engineering, radiation biology, and radiochemistry and nuclear chemistry. Due to practical considerations, this selection was limited; nonetheless, the authors emphasize the importance of other worker cohorts, including technologists who work in medical radiation therapy and imaging, and radiation epidemiologists and ecologists, who draw on the basic sciences of physics, chemistry, mathematics, and biology and play a significant role in radiation protection. It is hoped that these and other groups will be considered in future works.

The methods used to prepare this review included surveying relevant information on each workforce, using data from the literature and other resources, such as information from professional societies. All data were evaluated by teams of subject matter experts, comprised of leaders in each of the respective professions. However, it must be emphasized that some of the professions have few to no means of surveilling their workforce, particularly those that lack defined training programs. Under these circumstances, the writing teams relied on trends within associated professional or scientific societies, as well as their personal experience and observations. However, it must be plainly stated that the task of assessing the current status of the individual and overall professional radiation workforce involved a great deal of uncertainty. Much of this arose from incomplete (or nonexistent) surveillance data in some of the disciplines, precluding any precise assessment of status and temporal trends. Even among the professions where such data existed, frequently there was controversy over basic definitions, for example, the qualifications required to be considered as a professional health physicist.

Predicting the future outlook of professions proved challenging since it includes the need for baseline assumptions about future conditions; these are frequently upended by unforeseen events, for example, the Fukushima disaster and the Covid‐19 pandemic, with the latter in particular changing workforce environments and dynamics. Indeed, the review discusses several prominent reports that predicted shortages of health physicists that were not confirmed by subsequent studies. Nonetheless, given the expertise within each subcommittee, the team as a whole felt able to reach broad consensus opinions and recommendations, while identifying important issues that remain controversial and openly acknowledging the uncertainties. Overall, the authors conclude that the professions of Radiation Biology and radiochemistry and nuclear chemistry are operating with less‐than‐adequate workforces. Furthermore, the future outlook for all of the radiation professions assessed in this review appears mixed and uncertain. The factors underlying the observed workforce trends include shrinkage due to worker retirements without adequate replacements, a decline in the capacity of higher‐education pipelines, the closure of many training programs, and an overall decline in employment opportunities in some fields, limiting the attractiveness of those fields as a prospective career choice for incoming workers. These findings should act as a clarion call for action since the safe use of ionizing radiation is essential to meeting our nation's needs in health care, energy, homeland security, and defense.

Recommendations for each profession are provided in Chapters 2 through 7. Chapter 8 synthesizes and summarizes the findings from the profession‐specific chapters to form an overall impression of the status and outlook of the radiation professions individually and as part of the workforce as a whole.

## AUTHOR CONTRIBUTIONS

All the authors listed have contributed directly to the intellectual content of the manuscript. Wayne Newhauser and Jacqueline Williams drafted the manuscript.

## CONFLICT OF INTEREST

The authors declare that there is no conflict of interest that could be perceived as prejudicing the impartiality of the research reported.

## Data Availability

Data sharing is not applicable to this or the accompanying articles since no new data were created or analyzed during the generation of this workforce report.

